# *In Vivo* Long-Term Monitoring of Circulating Tumor Cells Fluctuation during Medical Interventions

**DOI:** 10.1371/journal.pone.0137613

**Published:** 2015-09-14

**Authors:** Mazen A. Juratli, Eric R. Siegel, Dmitry A. Nedosekin, Mustafa Sarimollaoglu, Azemat Jamshidi-Parsian, Chengzhong Cai, Yulian A. Menyaev, James Y. Suen, Ekaterina I. Galanzha, Vladimir P. Zharov

**Affiliations:** 1 Arkansas Nanomedicine Center, University of Arkansas for Medical Sciences (UAMS), Little Rock, Arkansas, United States of America; 2 Department of General and Visceral Surgery, University hospital of Frankfurt, Frankfurt am Main, Germany; 3 Department of Biostatistics, University of Arkansas for Medical Sciences, Little Rock, Arkansas, United States of America; 4 Department of Otolaryngology - Head and Neck Surgery, University of Arkansas for Medical Sciences, Little Rock, Arkansas, United States of America; King Faisal Specialist Hospital & Research center, SAUDI ARABIA

## Abstract

The goal of this research was to study the long-term impact of medical interventions on circulating tumor cell (CTC) dynamics. We have explored whether tumor compression, punch biopsy or tumor resection cause dissemination of CTCs into peripheral blood circulation using *in vivo* fluorescent flow cytometry and breast cancer-bearing mouse model inoculated with MDA-MB-231-Luc2-GFP cells in the mammary gland. Two weeks after tumor inoculation, three groups of mice were the subject of the following interventions: (1) tumor compression for 15 minutes using 400 g weight to approximate the pressure during mammography; (2) punch biopsy; or (3) surgery. The CTC dynamics were determined before, during and six weeks after these interventions. An additional group of tumor-bearing mice was used as control and did not receive an intervention. The CTC dynamics in all mice were monitored weekly for eight weeks after tumor inoculation. We determined that tumor compression did not significantly affect CTC dynamics, either during the procedure itself (*P* = 0.28), or during the 6-week follow-up. In the punch biopsy group, we observed a significant increase in CTC immediately after the biopsy (*P* = 0.02), and the rate stayed elevated up to six weeks after the procedure in comparison to the tumor control group. The CTCs in the group of mice that received a tumor resection disappeared immediately after the surgery (*P* = 0.03). However, CTC recurrence in small numbers was detected during six weeks after the surgery. In the future, to prevent these side effects of medical interventions, the defined dynamics of intervention-induced CTCs may be used as a basis for initiation of aggressive anti-CTC therapy at time-points of increasing CTC number.

## Introduction

Most breast cancer-related deaths are associated with metastases formed by CTCs [1–3]. In breast cancer-diagnostic procedures, the mammography and the punch biopsy are considered as gold standards [4,5]. However, it has been long discussed whether the tumor may be influenced during mammography, biopsy and surgery. Tumor damage may eventually result in shedding of additional CTCs into the blood system [6–11]. In particular, during the mammography, breast tissues are significantly compressed to improve image quality by increasing the contrast and reducing the scattered radiation of the breast tissue by decreasing the thickness. Therefore, the breast is compressed to a level just below the maximum setting of the machine (generally 200 N) which is close to the patient’s pain threshold [6,12]. The biopsy, performed in a significant proportion of cases, confirms a putative diagnosis of malignancy. However, this procedure locally damages tumor tissue and increases the potential for malignant cells to detach from the primary tumor, migrate into the adjacent soft tissue, intravasate into blood vessels and disseminate through the body, which eventually may lead to progression of metastatic disease [13]. Finally, it has long been suspected that surgery (one of the gold standard breast cancer-treatment procedures) can lead to increased dissemination of tumor cells [11,14–16].

Recently, using photoacoustic (PA) flow cytometry with focus on label-free detection of naturally light-absorbing CTCs in melanoma-bearing mice [17–20], we demonstrated that palpation, incisional biopsy, and partial tumor resection initiate CTC release into the bloodstream, leading to a dramatic (10-60-fold) increase in CTC counts above initial levels during and for two hours after the procedure. In contrast, complete resection of a primary melanoma tumor with tumor-free margin resulted in a rapid (few hours) disappearance of previously observed CTCs from blood [17,18]. However, very little is known about other cancer types and the long-term dynamics of CTCs during cancer progression. In addition, inoculation of tumor cells into a mouse ear in our previous studies is far from the clinical situation in breast cancer.

Here, the long-term influence of medical procedures on CTC dynamics in clinically relevant orthotopic xenograft mouse-tumor models is considered using real-time noninvasive CTC detection directly in the blood flow.

## Materials and Methods

### Principles of *in vivo* Fluorescence Flow Cytometry (FFC)

Real-time quantification of the CTCs in mouse blood was performed by means of *in vivo* FFC described elsewhere [21–23]. A 488-nm diode laser with a total power of 7 mW in the sample was used to excite fluorescence in CTC directly in blood vessels. A 40x objective (NA 0.75, Plan Fluor, Nikon) focused the laser beam into the blood vessel and to collect the excited fluorescence. Laser light and fluorescence were separated using dichroic mirrors. An additional cylindrical lens with a focal length of 250 mm shaped the laser beam in the sample into a line (5×50 μm). The fluorescence was collected through a band pass filter 520±15 nm (Semrock, Inc., Rochester, NY) and detected by a photomultiplier tube (PMT, R928, Hamamatsu, Co., Bridgewater, NJ). The PMT signal was acquired using a PCI-5124 digitizer (National Instruments, Austin, TX) and analyzed using a custom MatLab software (MathWorks, Natick, MA).

### Cell lines and animal model

This study was carried out in accordance with the recommendations in the Guide for the Care and Use of Laboratory Animals of the National Institutes of Health. The protocol was approved by the Institutional Animal Care and Use Committee of the University of Arkansas for Medical Sciences (Permit Number: 3389). All surgery was performed under anesthesia, and the efforts were made to minimize suffering.

The human breast cancer cell line MDA-MB-231-luc2-GFP (Caliper Life Sciences), which expresses Green Fluorescent Protein (GFP) and luciferase, was selected for this study. GFP expression was used as an intrinsic marker for CTC detection in blood circulation using *in vivo* FFC. The performance of the FFC method was previously verified using fluorescence microscopy of GFP-expressing CTCs in blood samples *in vitro* [18]. Luciferase-catalyzed bioluminescence was used as the marker for whole-body imaging of distant metastases.

The cells used in this study expressed GFP preferentially in cytoplasm. As a result of cell death, membrane rupture and formation of cell fragments leads to a dramatic decrease in cell fluorescence below the detection threshold of our *in vivo* FFC system. Additionally, small cell fragments generate the signal with lower amplitudes and narrower widths of signals. In this case it was possible successfully to distinguish whole, viable CTCs from GFP-labelled anucleate cell fragments in the ear artery [22].

Nude mice (Harlan Sprague–Dawley; weighing 20–25 g) were used as an animal model. To establish primary tumors in the orthotopic xenograft model of metastatic breast cancer, 5x10^6^ tumor cells in 5 μl of PBS were inoculated into a mammary gland. This model of human breast cancer was chosen because it is a well-established model of metastatic cancer in animal research and because the xenograft orthotopic model, which produces a primary tumor from human cells in a correct anatomical site, is most similar to human breast cancer.

### General experiment design

All tumor-bearing mice were separated into four groups including a control group (6 mice) and three treatment groups (6 mice per group) whose tumors were respectively manipulated by applying pressure on the tumor, by punch biopsy, or no-touch complete tumor resection two weeks after the tumor inoculation. Tumor diameters were measured in the long and short directions using a calibration ruler. The tumor volume was calculated as: Tumor volume = 1/2 (long diameter X short diameter^2^).

All mice were weekly analyzed using an IVIS200 Imaging System (Caliper Life Sciences) to detect the bioluminescence from the tumor and eventual metastasis after intraperitoneal injection of 30 μl Luciferin (Caliper Life Sciences). The number of CTCs in the blood circulation was enumerated *in vivo* using FFC under isoflurane anesthesia (inhalation, 1.2%). An anesthetized mouse was placed on a temperature-controlled microscope stage (37°C), with an ear spread flat over the glass window of the microscope. A small ear artery (typically 50 μm in diameter) was used to monitor the CTCs. All the mice underwent FFC monitoring under general anesthetic weekly for eight weeks after tumor inoculation. The duration of regular FFC monitoring was one hour on Week 1, three hours on Week 2, and one hour on Weeks 3–8 after tumor inoculation. Specifically, at Week 2, CTCs in the mouse groups that received a medical intervention were quantified one hour before the intervention (to collect the baseline CTC count), during the intervention, and two hours after the intervention was concluded. Results from the continuous monitoring of each mouse were binned into consecutive 10-minute windows, and the number of CTCs detected in each mouse during each window was counted.

### CTC dynamics in the control group

Six mice were inoculated with tumor cells, but did not receive tumor manipulation.

This group served as the control group to which the medical-intervention groups were compared for differences in the number of CTCs and CTC-release rates.

### Pressure-related CTC release

Two weeks after tumor inoculation, a weight (400 grams per 0.5 cm^2^ area) was applied directly to the tumor for 15 min in six mice. The pressure level was set using a digital pressure sensor (Loadstar Sensor, DI-100) to mimic the pressure experienced during a mammography.

### Punch biopsy-related CTC release

A punch biopsy was conducted in six mice using a 2.0 mm biopsy punch with a plunger system (BPP-20F 2.0 mm w/plunger, Miltex, Inc) two weeks after tumor inoculation.

### Surgery-related CTC release

CTC release was analyzed in six mice during surgery. After one hour of continuous CTC monitoring (before surgery), the skin of the mouse’s mammary gland was washed twice with chlorihexidine and allowed to dry for three minutes. Complete tumor resection was conducted by cutting the tissue outside the 1-to-2-mm tumor margin. Each resection took ~ 2 minutes. The remaining tissue was stitched closed (Suture: Non-absorbable, size: 3.0, Ethicon Inc). Skin sutures were removed seven days after the surgery.

### Statistical analysis

Each of the four treatment groups were analyzed separately. Within a treatment group, each mouse was subjected weekly to at least 60 minutes of *in vivo* monitoring for CTCs, and the CTC detection rate (in CTCs per minute) was determined. The resulting data were analyzed for changes from week to week via Poisson regression with generalized estimating equations to accommodate the non-independence between measurements made at different weeks on the same mouse. For the Biopsy, Pressure, and Tumor Control groups, data were analyzed via Poisson regression with generalized estimating equations to accommodate the non-independence between measurements made before, during, and after manipulation on the same mouse. For the Surgery group, however, Poisson regression could not be used because of complete CTC disappearance from all mice afterwards, and the change in CTC counts after surgery were analyzed instead via the signed-rank test. All comparisons employed an alpha = 0.05 significance level, and all statistical analyses employed SAS v9.3 software (The SAS Institute, Cary, NC).

## Results

### CTC dynamics, tumor growth and development of metastasis in tumor-bearing mice without interventions (control)

**Fig 1:**
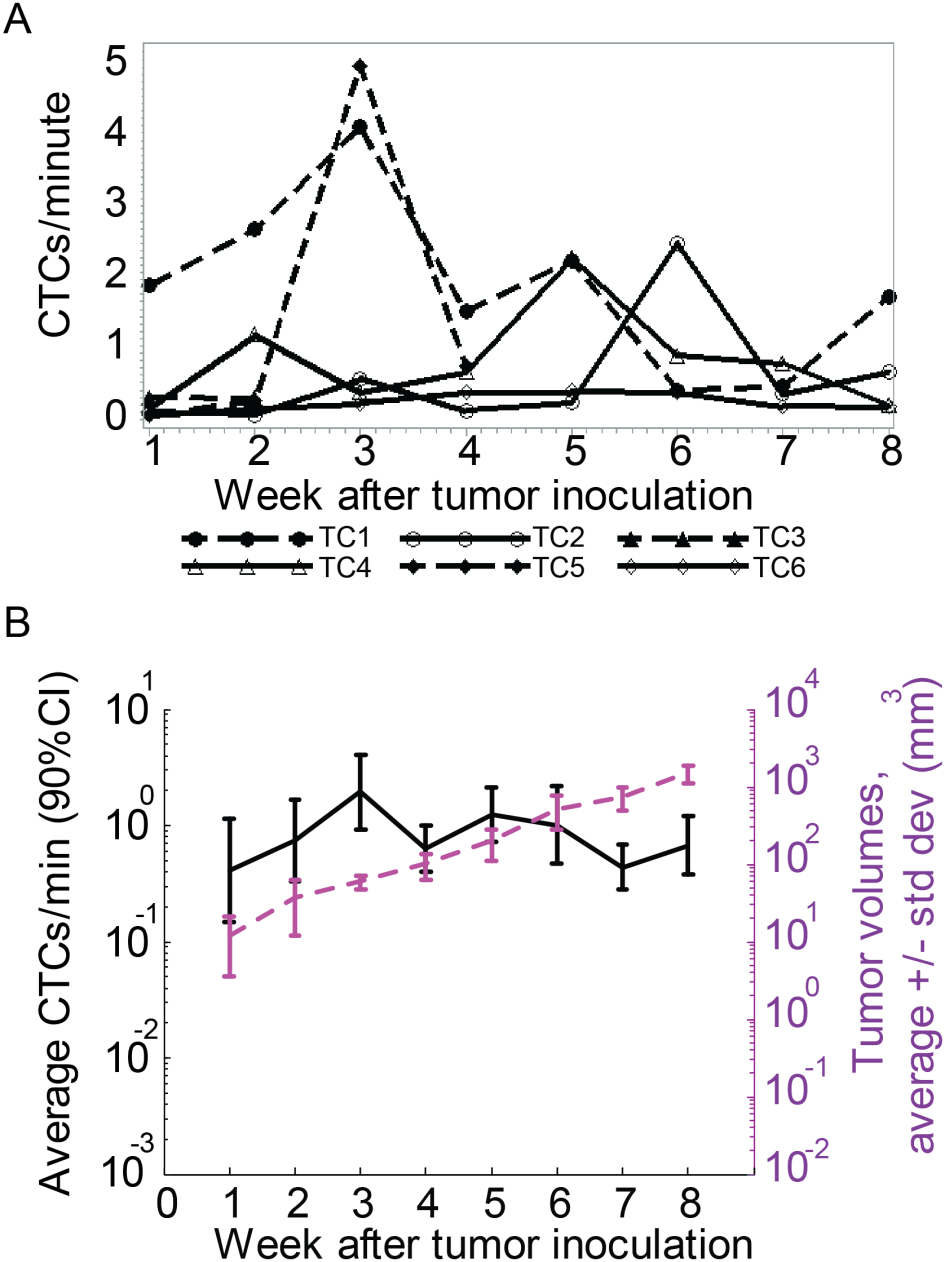
CTC dynamics during eight weeks in tumor control mice. (A) Profile plot of CTC detection rates (in CTCs/minute) measured weekly from six different mice inoculated with breast cancer cells (MDA-MB-LUC2-GFP) during the eight weeks after tumor inoculation. (B) Profile plot of the average number of CTC signals per minute and average tumor volume during the same eight weeks. Values and error bars represent the averages and SDs of CTC counts from n = 6 mice.

The control group of breast cancer tumor-bearing mice was used to identify the normal rate for CTC release by the tumor. On average, the CTC count varied from 0.5 to 2 cells/min during 8 weeks of tumor growth. As we previously reported [18], the CTC count fluctuated and did not strongly correlate with the tumor size. On average, the maximal CTC concentration was observed three and five weeks after tumor inoculation (Fig 1).

### Changes in CTC dynamics after application of pressure to primary tumors

**Fig 2:**
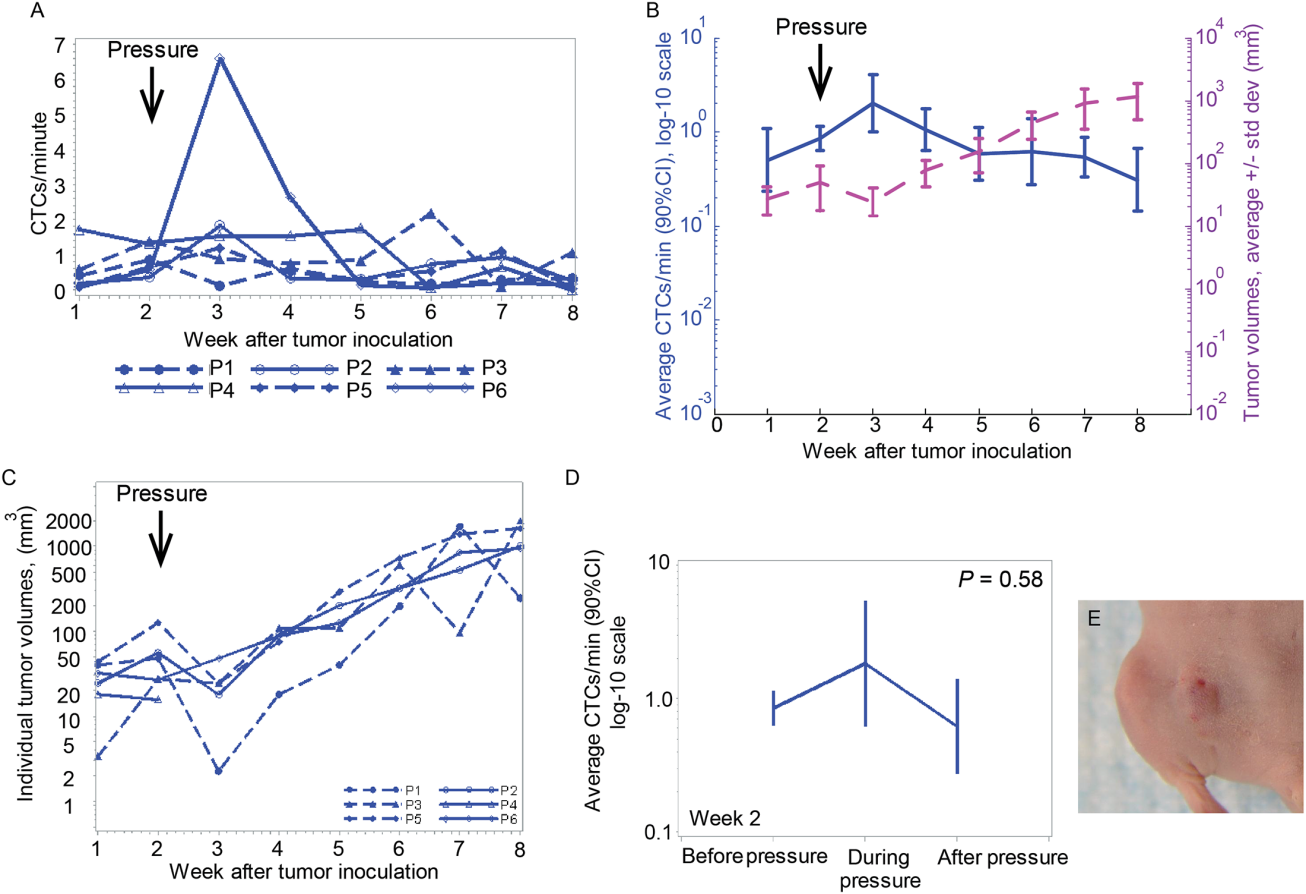
CTC dynamics change after tumor pressure. (A) Profile plot of CTC detection rates (in CTCs/minute) measured weekly from six mice inoculated with breast cancer cells (MDA-MB-LUC2-GFP) during the eight weeks after tumor inoculation. Pressure was applied at week 2. (B) Profile plot of the average number of CTC signals per minute and average tumor volume during the same eight weeks. Values and error bars represent the averages and SDs of CTC counts from n = 6 mice. (C) Individual tumor volumes from six mice after tumor inoculation. Pressure was applied at week 2 after tumor inoculation. (D) Profile plot of average number of CTC signals per minute from 60 min before, 15 min during, and 120 min after removing pressure provided by a cylindrical 400 g weight with 10-mm diameter. (E) Image of the tumor after 15 minutes of compression using digital pressure-controller software (Loadstar Sensor, DI-100).

The average CTC dynamics for mice receiving a weight to the tumor were close to those in the control-group mice. The maximal CTC count was observed on Week 3 (Fig 2A and 2B) and varied in the range 0.5–2 cells/min, similar to that seen in the control group. Noticeably, the tumor size decreased after applying weight on the tumor and re-increased again two weeks later (Fig 2B, 2C and 2E). In one case (P4) the tumor size decreased after applying the weight to the point that tumor were not identified by whole body imaging, but CTCs were still detected (Fig 2A and 2C).

A comparison of CTC counts at week two, before the pressure was applied (0.9 cells/min), during the pressure application (1.9 cells/min), and after the pressure was released (0.6 cells/min) shows that application of pressure had little impact on the number of CTCs (Fig 2D). Specifically, the CTC count increased 2.11 folds and reached the maximum during manipulating the tumor, but the increase was not statistically significant (*P* = 0.28). Moreover, after pressure removal, the average CTC rate fell to 74% of the level it had before the manipulation, but this decrease was also not significant (*P* = 0.59), and during six weeks afterwards, no difference in CTC dynamics between this group of mice and the tumor control mice group was observed.

### Punch biopsy*-*related changes in CTC dynamics

**Fig 3:**
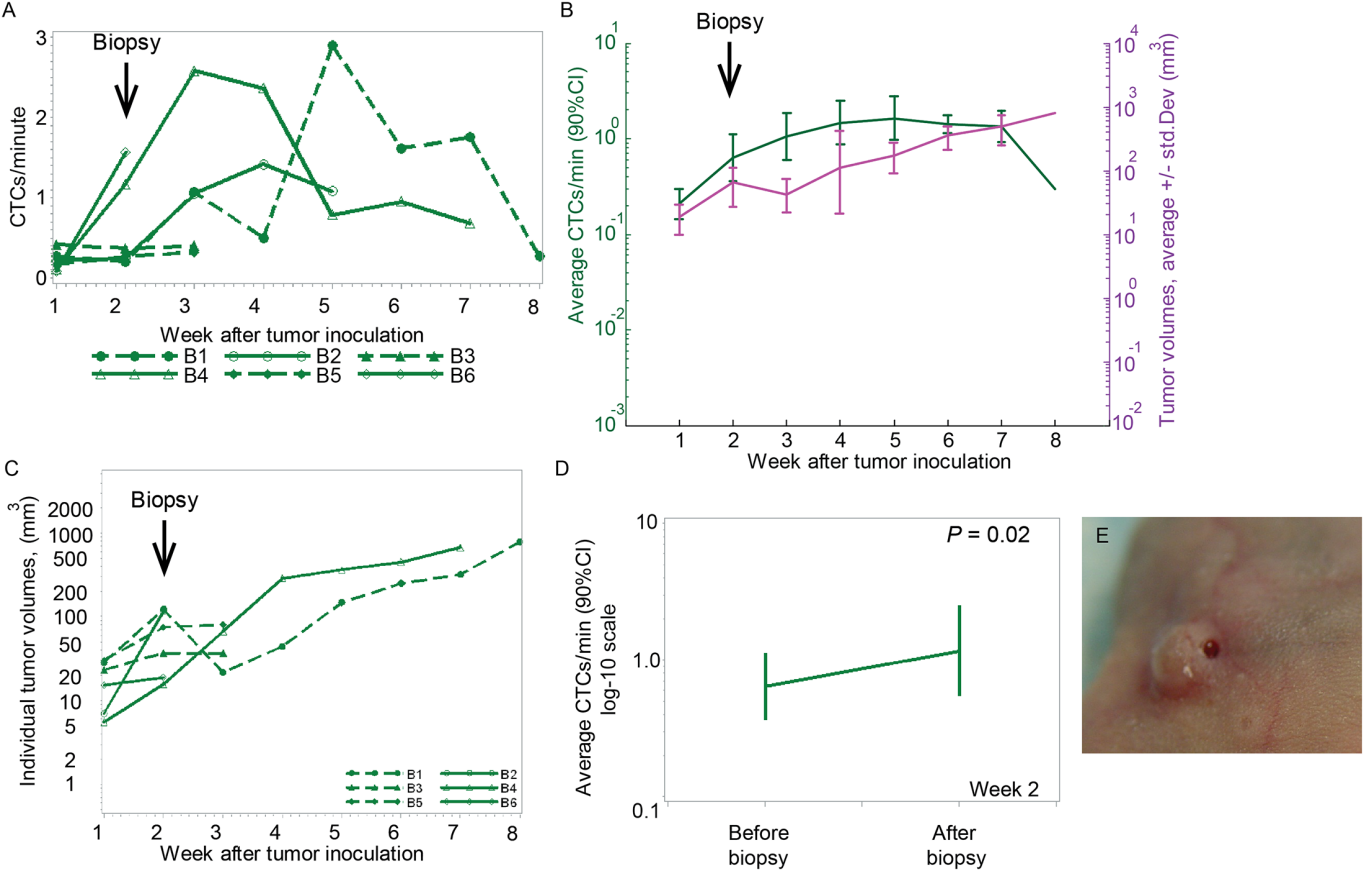
Change in CTC dynamics after punch biopsy. (A) Profile plot of CTC detection rates number of CTCs per a minute measured weekly from six mice inoculated with breast cancer cells (MDA-MB-LUC2-GFP) during the eight weeks after tumor inoculation. Punch biopsy was performed at week 2 after tumor inoculation. (B) Profile plot over time of the average number of CTC signals per minute and average of the tumor volume during the same eight weeks. Values and error bars represent the averages and SDs of CTC counts from n = 6 mice. The average of CTC rate per 8 weeks was calculated for five mice at week 3, three mice at week 5 and two mice at week 7. (C) Individual tumor volumes from six mice. Punch biopsy was performed at week 2 after tumor inoculation. (D) Profile plot of the average number of CTC signals per minute from 60 min before and 120 min after the punch biopsy, showing a 1.82-fold increase in the CTC-detection rate (P = 0.02). (E) Image of the tumor after a punch biopsy.

Compared to control group, we observed a steadier rate of CTC detection after the biopsy for six weeks afterward (Fig 3A and 3B). During the 8-week experiment, the CTC detection rate in this group of mice varied from 0.2–1.6 cells/min (Fig 3B). The punch biopsy performed on a tumor two weeks after inoculation did not affect the tumor volume in five mice. The tumor volume increased continuously during the eight weeks after tumor inoculation (Fig 3B, 3C and 3E).

At week two, the punch biopsy immediately caused a significant increase in the number of CTCs. The baseline CTC detection rate determined for 1h before the biopsy (0.64 cells/min) was elevated 82% to the level of 1.17 cells/min for the two hours after biopsy ended (*P* = 0.021; Fig 3D). Given the short duration of the biopsy, it was impossible to determine the actual increase in the number of CTCs “during” the procedure.

### Change in CTC dynamics after a complete tumor resection

Surgery was conducted on six mice two weeks after tumor inoculation. Histology exams performed on the resected tumor tissues confirmed that, for 5 mice out of 6, there was no residual tumor left and tumor-free margins were achieved.

**Fig 4:**
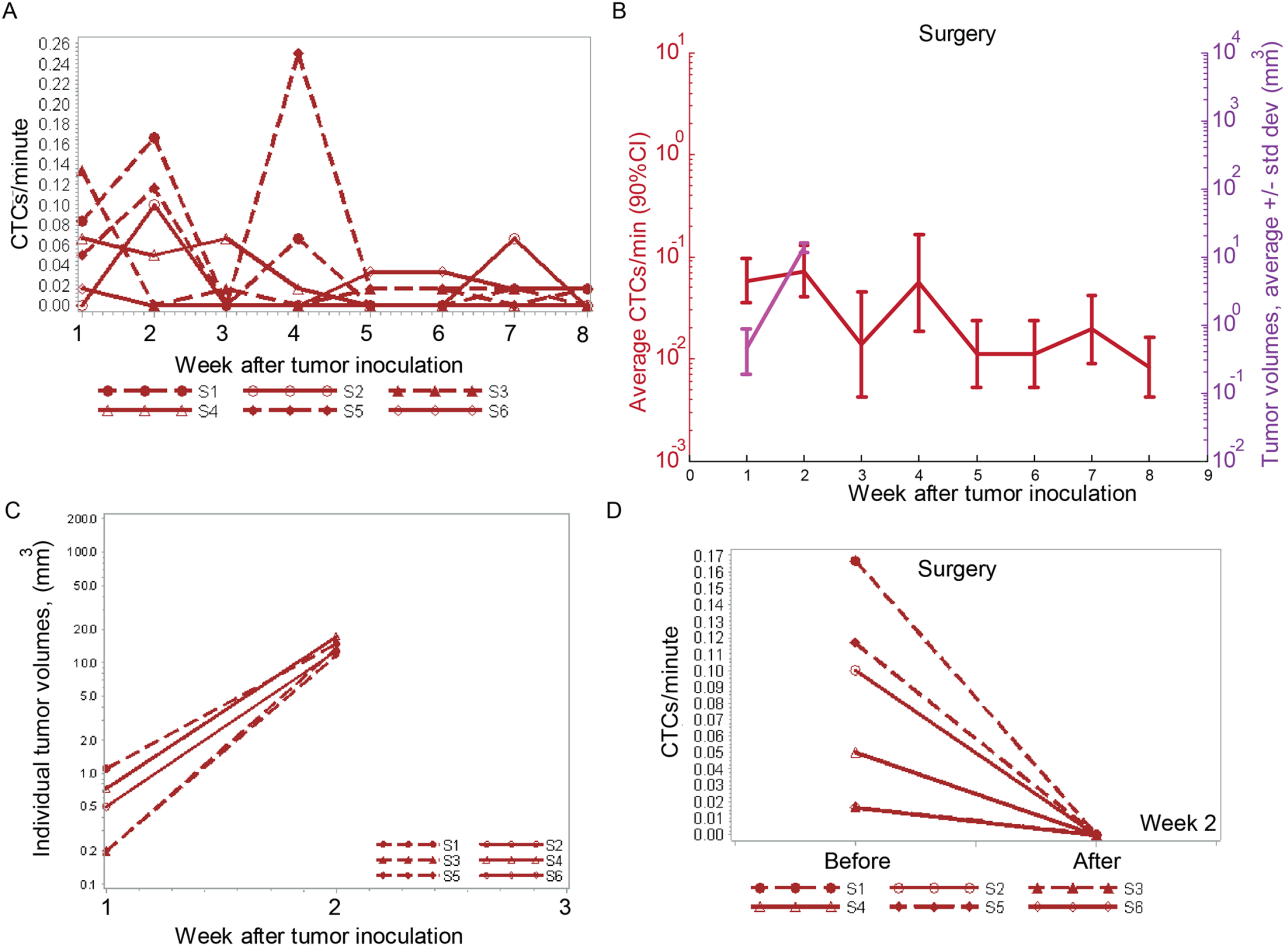
Change in CTC dynamics after surgery. (A) Profile plot of CTC detection rates (in CTCs/minute) measured weekly from six mice inoculated with breast cancer cells (MDA-MB-LUC2-GFP). Surgery was conducted at Week 2 after tumor inoculation. (B) Profile plot of the average number of CTC signals per minute and average tumor volume during eight weeks from n = 5 mice after excluding the mouse which received partial tumor resection. Values and error bars represent the averages and SDs of CTC counts from n = 5 mice. (C) Individual tumor volumes from six mice. Surgery was performed at week 2 after tumor inoculation. (D) Profile plot of the average number of CTC signals per minute from 60 min before and 120 min after the surgery.

Compared to the control group, a dramatic decrease in the number of CTCs was observed from 0.1 cells/minute at week two to ~0.01 cells/minute at weeks three-to-eight. (Fig 4A and 4B). However, CTC recurrence in small numbers was detected during five weeks after the surgery (Fig 4A and 4B).

In all the mice, no tumor visibly re-grew during the six weeks after tumor resection (Fig 4C).

A short-term effect of complete tumor resection was even more profound. During the one hour long CTC monitoring “before” the tumor resection, a total of 28 CTCs were detected among the 6 mice, yielding an average CTC detection rate of 0.08 cell/minute. During and for two hours after the tumor resection, no CTCs were observed (*P* = 0.03) (Fig 4D).

**Fig 5:**
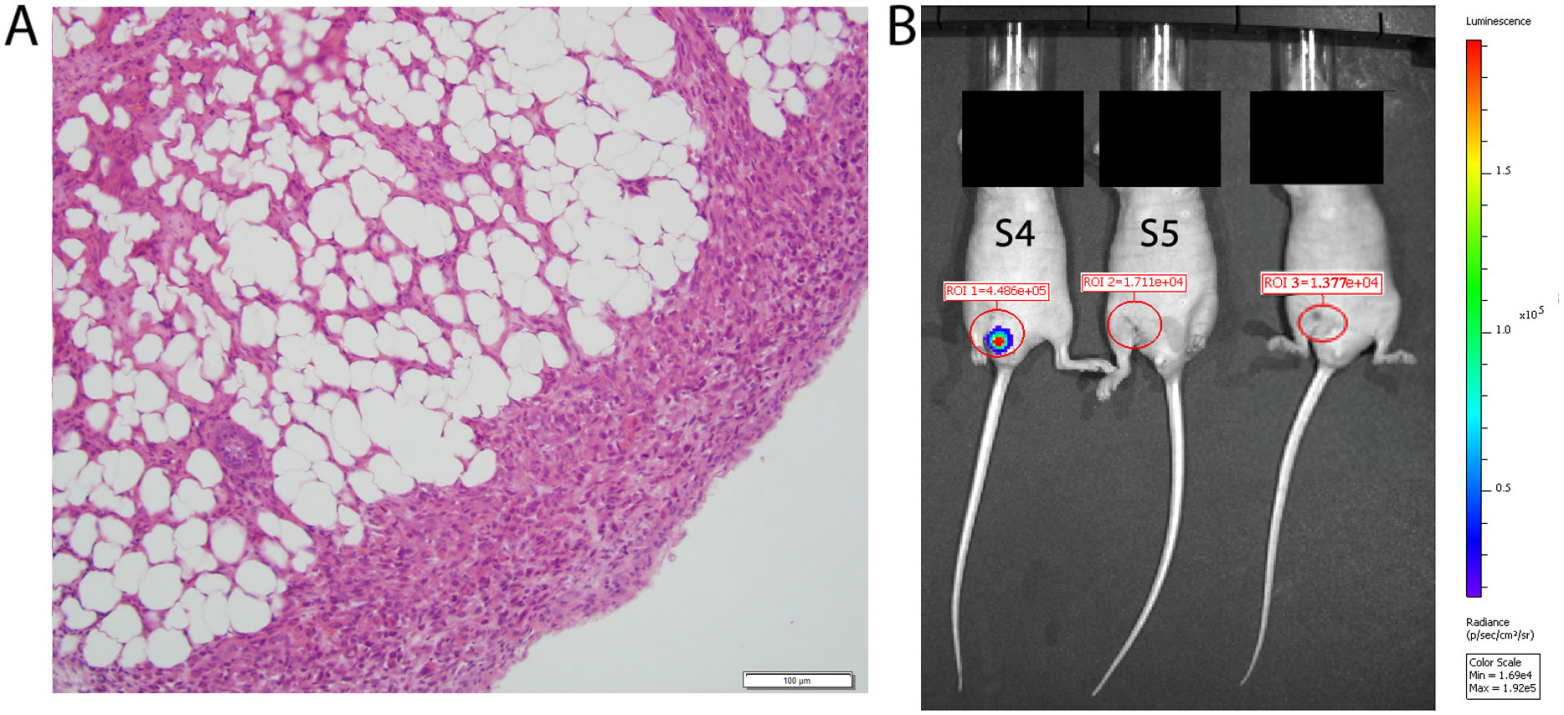
Tumor-positive margin and IVIS image after partial tumor resection. (A) Histological Image of the resected tumor from mouse S5 with tumor-positive margin. (B) IVIS image of three different mice two weeks after receiving a tumor resection. The IVIS image from mouse S4 shows a positive bioluminescent signal in the previous tumor area (ROI 1) two weeks after receiving a tumor resection. In this mouse, the histological exam showed tumor-free margin from the resected tumor. The IVIS image of positive bioluminescent signal in the previous tumor area near the tail is due to skin shifting after stitching. In the IVIS image of mouse S5 no residual tumor was observed two weeks after receiving a tumor resection but the histology exam of the resected tumor showed a positive tumor margin.

We should note that, for one mouse (S5), the histology exam of the resected tumor revealed a positive tumor margin (Fig 5A). The CTC count for this mouse was the highest in the group for six weeks after the surgery, with the maximal CTC concentration observed two weeks after the surgery. Whole-body imaging in both fluorescence and bioluminescent signals, no residual tumor was observed (Fig 5B). For another mouse (S4) the histology exam confirmed tumor-free margins; however the level of bioluminescence (IVIS system) for this mouse was increased at the surgery site two weeks after the procedure (Fig 5B). The CTC detection rate for this mouse was elevated one week after the surgery, but it decreased later on (Fig 4A).

## Discussion

The CTC count provided certain insights on the status of metastatic tumor development; however, in this work we focused on the long-term changes in the number of CTCs that could be associated with medical intervention for diagnostic or therapy purposes.

Our study confirmed that diagnostic and surgical procedures may trigger a release of intervention-induced CTCs for long-term in the breast cancer-bearing mouse model. We assessed CTC dynamics during eight weeks under impact of pressure, punch biopsy and complete primary tumor resection as widely practiced and obligatory diagnostic and therapeutic interventions applicable to breast cancer patients. Punch biopsy in our murine xenograft animal model led to an average 1.82-fold increase in the CTC rate after performing the procedure (*P* = 0.02), and the dynamics of CTCs stayed elevated up to five weeks after the biopsy. These results are in line with our previous findings in melanoma-bearing mice [17]. However, the incisional biopsy led to an increase in CTC rate during the biopsy and immediately afterwards. These differences can be explained in part by damage to the intratumor vascularity after an incisional biopsy, which was greater than after a punch biopsy.

Differences between breast cancer and melanoma were observed after applying pressure on the tumor. The compression on the melanoma tumor led to a significant increase in the CTC rate for two hours after compression removal [17]. The compression on the breast cancer did not significantly affect CTC dynamics during the compression procedure itself. Additionally, during six weeks after compression removal, no difference in CTC dynamics was observed between this group of mice and the tumor-control group, which did not receive any medical intervention. These findings are consistent with recently published data on *ex vivo* quantification of CTCs in breast cancer patient blood before and after mammography [6]. We note here that Fornivik, et al did not determine the CTC rate during the application of the compression to the tumor.

Our data indicates that radical breast cancer surgery indeed removes the main source of CTCs. After complete tumor resection, CTC counts were dramatically decreased close to zero in all the mice (*P* = 0.03) and did not increase during six weeks afterwards. This confirmed our initial assumption that the major bulk of CTCs originate from a primary tumor. Moreover, this is further confirmed by the discovery of an increased CTC count (compared to other mice from the same group) for a mouse having incomplete tumor resection. Based on these results, we suggests that real-time monitoring of CTC levels *in vivo* during surgery and long-term afterwards can be used as a new biomarker of surgery efficacy: stable disappearance or significant decrease of CTC number may be associated with negative tumor margins while significant amount of residual CTCs may indicate positive tumor margins. However, more research on this topic is required to confirm the association between positive tumor margins and CTC dynamics.

Finally, our CTC-count data indicates that the number of CTCs is decreased after complete tumor resection. However, CTCs were later discovered in 80% of mice but the CTC rate was detected in lower level than before the procedure. It is possible that metastases may be formed and led to CTCs release after tumor resection. Again, more research on this topic is required to confirm this hypothesis.

Such rare cells or distant metastases might contribute to disease relapse observed in patients treated with surgery alone with a peak in recurrences 2–4 years after surgery and later, more extended increases in metastatic disease between 6–15 years after removal of the primary tumor [24,25]. The valuation of breast cancer as a systemic disease led to the attempt to eliminate the residual CTCs using additional therapy such as radiation and chemotherapy. Indeed, this is highly successful and leads to considerably improved outcome of patients with breast cancer [24,26], even after 30 years of follow-up.

## Conclusions

In conclusion, we present preclinical evidence that medical procedures such punch biopsy could enhance penetration of cancer cells from a primary tumor into the blood circulation in a murine xenograft breast cancer model. However, tumor compression did not change the long-term CTC dynamics, and after complete tumor resection, there was decreased tumor cell penetration into the blood flow or complete CTC disappearance for long-term. Our results have showed that *in vivo* flow cytometry could be used for early medical intervention-induced CTCs and to determine the long-term therapy efficacy.
